# Effects of Mountain Pine Beetle on Fuels and Expected Fire Behavior in Lodgepole Pine Forests, Colorado, USA

**DOI:** 10.1371/journal.pone.0030002

**Published:** 2012-01-17

**Authors:** Tania Schoennagel, Thomas T. Veblen, José F. Negron, Jeremy M. Smith

**Affiliations:** 1 Institute of Arctic and Alpine Research, University of Colorado, Boulder, Colorado, United States of America; 2 Department of Geography, University of Colorado, Boulder, Colorado, United States of America; 3 Rocky Mountain Research Station, United States Forest Service, Fort Collins, Colorado, United States of America; Lakehead University, Canada

## Abstract

In Colorado and southern Wyoming, mountain pine beetle (MPB) has affected over 1.6 million ha of predominantly lodgepole pine forests, raising concerns about effects of MPB-caused mortality on subsequent wildfire risk and behavior. Using empirical data we modeled potential fire behavior across a gradient of wind speeds and moisture scenarios in Green stands compared three stages since MPB attack (Red [1–3 yrs], Grey [4–10 yrs], and Old-MPB [∼30 yrs]). MPB killed 50% of the trees and 70% of the basal area in Red and Grey stages. Across moisture scenarios, canopy fuel moisture was one-third lower in Red and Grey stages compared to the Green stage, making active crown fire possible at lower wind speeds and less extreme moisture conditions. More-open canopies and high loads of large surface fuels due to treefall in Grey and Old-MPB stages significantly increased surface fireline intensities, facilitating active crown fire at lower wind speeds (>30–55 km/hr) across all moisture scenarios. Not accounting for low foliar moistures in Red and Grey stages, and large surface fuels in Grey and Old-MPB stages, underestimates the occurrence of active crown fire. Under extreme burning conditions, minimum wind speeds for active crown fire were 25–35 km/hr lower for Red, Grey and Old-MPB stands compared to Green. However, if transition to crown fire occurs (outside the stand, or within the stand via ladder fuels or wind gusts >65 km/hr), active crown fire would be sustained at similar wind speeds, suggesting observed fire behavior may not be qualitatively different among MPB stages under extreme burning conditions. Overall, the risk (probability) of active crown fire appears elevated in MPB-affected stands, but the predominant fire hazard (crown fire) is similar across MPB stages and is characteristic of lodgepole pine forests where extremely dry, gusty weather conditions are key factors in determining fire behavior.

## Introduction

Epidemic outbreaks of native mountain pine beetle (*Dendroctonus ponderosae*; MPB) populations have affected over 1.6 million ha of predominantly lodgepole pine (*Pinus contorta* var. *latifolia*) forests in Colorado and southern Wyoming since 1996. Policy makers, forest managers, and the public are concerned that resulting tree mortality will increase fire risk (probability of fire occurrence) and fire hazard (amount and configuration of flammable fuels, and resulting fire behavior), threatening communities in the wildland-urban interface, key watersheds, and recreation-based tourism for decades to come.

Mountain pine beetle and wildfire are the two primary disturbance agents in lodgepole pine forests. Both have increased significantly in recent years, especially in mid- to high-elevation forests of the central and northern Rockies [1], [2]. Under endemic conditions MPB typically kill larger, older trees weakened by drought or disease [3]. Under epidemic conditions MPB mount mass attacks that overwhelm vigorous trees and can result in high mortality of host species across thousands of hectares [4], [5]. The most recent MPB outbreaks have been linked to warmer and drier conditions and to past history of fire or land use that have promoted an abundance of older, large-diameter lodgepole pine trees, which increase susceptibility to the insect [6], [7], [8]. Infrequent high-severity fires associated with severe drought and high winds are characteristic in lodgepole pine forests, creating broad-scale age mosaics across the landscape. Drought conditions conducive to large wildfires in these high-elevation forests in Colorado are rare, typically recurring at >100- year intervals within a stand, and historically coinciding with the negative phases of the El Niño Southern Oscillation (La Niña) and Pacific Decadal Oscillation, and the positive phase of the Atlantic Multidecadal Oscillation [9].

In Colorado, the area of the current MPB outbreak has also experienced a significant increase in residential communities, with large areas of developed private lands adjacent to fire-prone public lands [10]. Therefore, understanding the effect of MPB mortality on subsequent wildfire risk and behavior is key for management of lodgepole pine forests in Colorado and elsewhere throughout the West, when specific objectives need to be met.

MPB affect potential fire risk and hazard through initial tree mortality, which alters the arrangement, composition, moisture content of forest fuels, and microclimate over time [11]. Temporal variation in the fuels complex is associated with distinct phases following the outbreak and with hypothesized changes in potential fire behavior.

In the initial stage following an outbreak, often called the Red stage, needles on trees killed by MPB experience a change in color from green to yellow to red 1–2 yrs after attack, but can take longer at higher elevations. Yellow and red needles experience a significant decrease (10 times) in foliar moisture content compared to green needles before they fall, which typically occurs 2–3 yrs after attack [12]. Page and Jenkins [13] and Hoffman [14] indicate a high probability of active crown fire during the Red stage, while Simard et al. [15] and Klutsch et al. [16] predicted that passive fire (surface fire with torching of individual crowns), rather than active crown fire through the canopy, was more probable during this Red stage and up to seven years after the outbreak.

After red needles have fallen from attacked trees to the forest floor, the stand enters the Grey stage, which lasts about 4–10 yrs after attack. This stage is characterized by standing beetle-killed trees with no foliage and some loss of smaller branches; larger dead fuels remain in the canopy, yet some beetle-killed trees or portions of their crowns have fallen to the ground. Crown fire hazard is hypothesized to fall below pre-outbreak levels due to loss of available crown fuels; with less fuel to burn in the canopy and greater spacing between tree crowns, the probability of active crown fire is expected to be lower [11], [15], [16]. More-open canopies provide less sheltering, however, which increases the wind speed within the stand and promotes drier surface fuels [17], while surface fuel loads may be higher due to fall of dead trees and canopy fuels.

Following the Grey stage is the Old-MPB stage, during which the majority of the standing snags from beetle-caused mortality fall, along with the release of trees and seedlings from the understory of the beetle-killed overstory, which provide important ladder fuels. Both Page and Jenkins [13] and Simard et al. [15] hypothesize an increase in active or passive crown fire potential relative to earlier stages, due to lower canopy bases and higher canopy bulk density, while Klutsch et al. [16] indicate a decrease in active crown fire potential in the equivalent Red, Grey, and Old stages compared to uninfested stands. Klutsch et al. [16] incorporated the contribution of fallen snags to potential fire behavior, however Page and Jenkins [13] and Simard et al. [15] did not consider this in their assessment of transition to crown fire due to modeling limitations.

A variety of surface-crown fire spread models such as BehavePlus, NEXUS, FFE-FVS, FlamMap and FARSITE integrate sub-models of surface fire behavior, transition to crown fire, and crown fire spread rate, based on Rothermel's surface [18] and crown [19] fire spread rate equations and Van Wagner's [20] crown fire initiation and spread equations. In general, operational fire behavior models assume surface and crown fuels are spatially homogeneous and continuous, with no explicit modeling of different mechanisms of heat transfer or transitory fire behavior, which may play important roles in fire behavior [14], [21]. Recently developed physics-based fire behavior models can address variability in fuels, and important fire-atmosphere interactions, making them well-suited for modeling potential fire behavior in stands affected by MPB-caused mortality, as explored by Hoffman [14]. Surface fuels in areas of MPB-caused mortality will vary spatially and lack continuity as a result of differential mortality levels across stands and landscapes. However, such modeling approaches are very computationally demanding and therefore limited in application. For example, while Hoffman et al. [14] explored the effects of many important fuel characteristics in MPB-affected forests on potential fire behavior, they were not able to consider the effect of variation in wind speed, which plays a fundamental role in the behavior of wildfire.

While none of the existing operational fire models were designed to predict fire behavior in stands affected by insect mortality, of these, BehavePlus [v. 5.0.4; 22] appears to be best suited for this purpose, as it can account for two important fuel characteristics in MPB-affected stands: 1) low available canopy fuel moistures in the Red and Grey stages and 2) high 1000-hr surface fuel load in Grey and Old-MPB stages when MPB-killed trees fall to the ground. Using this model, we predicted potential fire behavior across a gradient of wind speeds and three moisture scenarios in unattacked Green stands compared to three stages following initial MPB attack (Red [1–3 yrs post-attack], Grey [4–10 yrs post-attack], and Old-MPB [30 yrs post-attack]), based on empirical fuels data from lodgepole pine forests in Colorado.

## Methods

### Study Area

**Figure 1:**
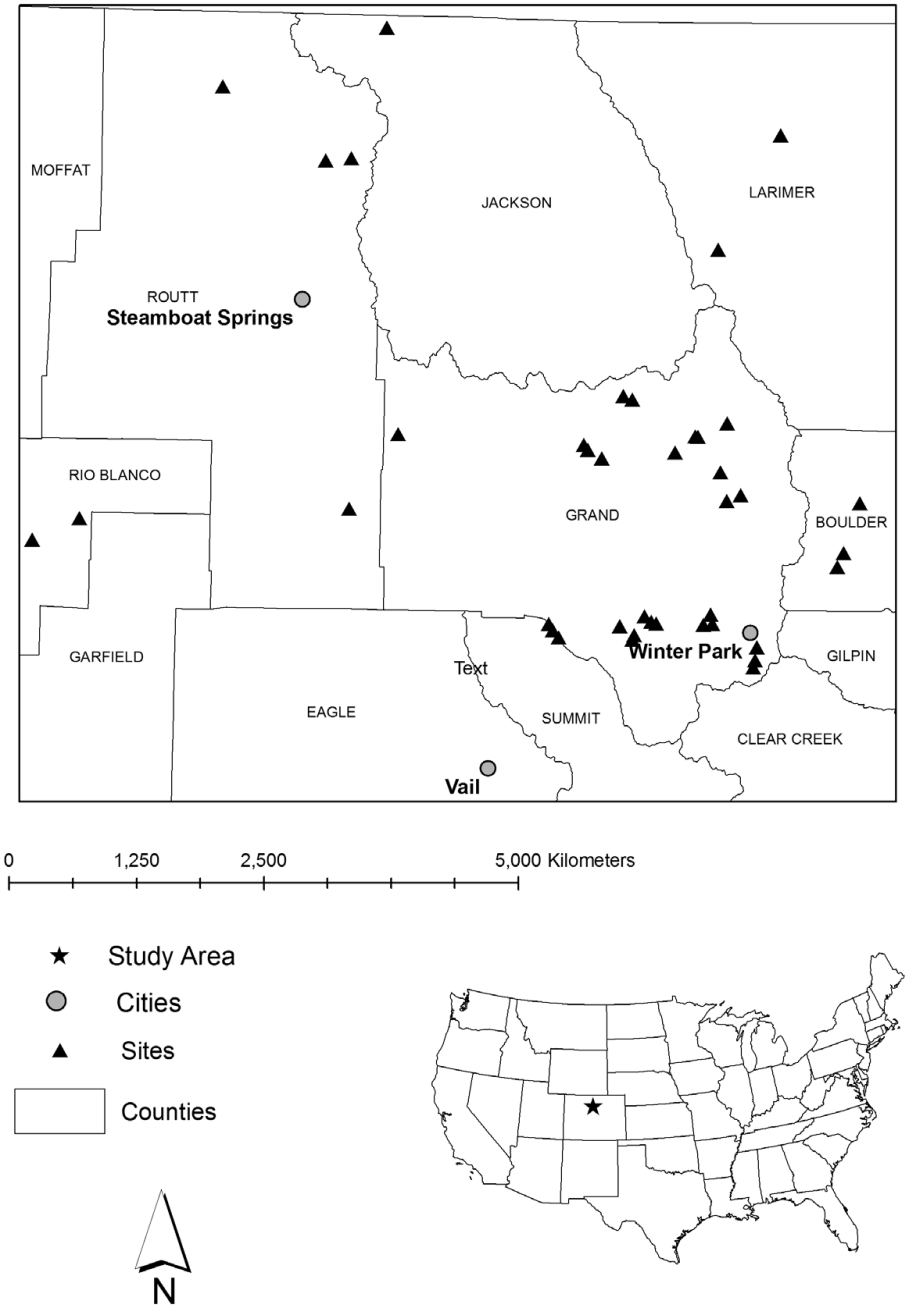
Study area map. Map of the study area in west-central Colorado, showing 40 sites sampled in four stages of time-since MPB attack.

The study area encompasses lodgepole pine forests of northern Colorado spanning the White River, Routt and Arapaho-Roosevelt National Forests between 2500 and 3200 m (Figure 1). Lodgepole pine forests vary from monospecific even-aged post-fire stands to heterogeneous stands where it co-occurs with Engelmann spruce (*Picea engelmanii*), subalpine fir (*Abies lasiocarpa*), and aspen (*Populus tremuloides*). In addition to the current outbreak, portions of the study area also experienced past MPB activity in the mid-1980s [23], [24].

### Field sampling

**Figure 2:**
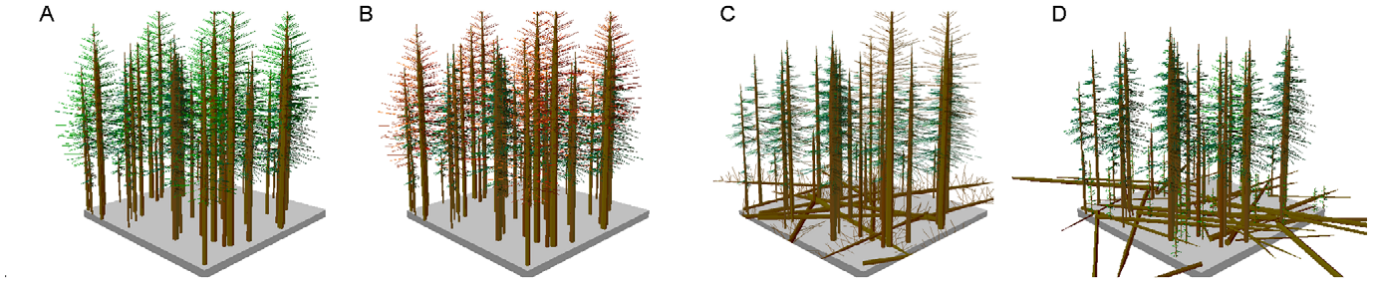
Idealized progression of four stages of MPB attack. Graphic characterizing an idealized sequence of Green unattacked stands, compared to the three stages subsequent to MPB attack. In this chronosequence, 40% of the trees were killed by MPB in the Red stand. In the Grey stand needles fall from the MPB-attacked trees with some attacked trees fallen, opening up the canopy and allowing for higher wind speeds. In the Old-MPB stand most of the MPB-attacked trees have fallen to the ground contributing to high 1000-hr surface fuel load and slightly diminished wind speeds compared to the Grey stand.

In 2007–2008 we sampled sites across the study area in each of four stages of MPB occurrence: 1) stands with no evidence of significant MPB activity in the last 50 yrs (Green stage, though some recently attacked trees were present, characteristic of endemic levels of MPB), 2) stands attacked by MPB in the last 3 yrs (Red stage), 3) stands affected by MPB 4–10 yrs ago, where the majority of MPB-attacked trees have dropped their red needles, but remain standing (Grey stage), and 4) stands affected by MPB ∼30 yrs ago, where the majority of MPB-affected trees are no longer standing (Old-MPB stage; Figure 2). Many of the Old-MPB stands were affected to some degree by the current outbreak (i.e. presence of red trees), so in order to remove the confounding effect of the current outbreak on potential fire behavior of the Old-MPB stands, we considered red trees in these stands as green trees for modeling purposes. Sampling in 2007 included 17 sites (4 Green, 8 Red and 5 Grey 0.02-ha stands) and in 2008 included 23 sites, where we subsampled each site (three stands [subplots] of 0.01 or 0.04 ha depending on tree density) and averaged across subplots for site values, totaling 10 sites in each of four MPB stages (n = 40; Figure 1). All necessary permits were obtained for the described field studies from the U.S Forest Service.

To determine the timing of prior (pre-1997) MPB mortality in Old-MPB stands [25] we cored trees according to two criteria, where: 1) fallen or standing dead lodgepole pine showed evidence of prior MPB-caused mortality such as galleries or blue stain (∼20 trees/stand), and 2) fir and spruce, which are not MPB-hosts, or live lodgepole trees within 4 m of lodgepole trees killed by prior MPB attack, could exhibit growth releases corresponding with presumed timing of the prior MPB mortality. We also cored mature live or recently killed lodgepole pine to determine stand origin. This protocol resulted in an average of 40 trees cored/stand.

In the lab, cores from Old-MPB stands were mounted and sanded according to standard practices [26]. When core samples did not include the pith, a geometric model of annual tree growth estimated the number of missing rings to the pith [27]. A subset of cores, based on soundness of wood, was selected for measurement and subsequent crossdating analysis [28], using a site-based regional chronology of lodgepole pine (e.g. Cameron Pass; [29]). Based on correlation of host-tree death dates and non-host tree growth releases [30], the approximate date of prior MPB attack was estimated for each Old-MPB stand [25].

#### Surface fuel sampling and calculations

Surface fuels were sampled following methods by Brown [31], according to fuel size and type. Dead surface fuels were categorized by the time lag needed for a fuel particle of given diameter to equilibrate with ambient relative humidity given static weather conditions. At each stand (0.01, 0.02, 0.04 ha), we sampled surface fuels along two perpendicular 20-m transects by counting the number of transect intersections by time lag fuels: 1) 1-hr (<0.6 cm diameter) and 10-hr (0.6–2.5 cm diameter) from 0–2 m along the transect, 2) 100-hr (2.5–8 cm diameter) pieces from 0–5 m, and 3) 1000-hr (>7.6 cm) pieces from 0–20 m; the diameter and decay class of 1000-hr pieces were also recorded. At 10 m and 18 m along each transect, we measured the depth of the fuelbed, litter and duff, and established 2-m diameter vegetation microplots, where we estimated height (to nearest 10 cm) and assigned one of five percent cover classes of live/dead shrubs, herbs, and grasses; and estimated the cover of litter and bare ground.

Surface fuel loads were calculated using the following protocols. Dry-weight biomass of understory vegetation (live/dead shrubs, herbs, and grasses) was estimated by calculating the mid-point of the percent cover class*height*bulk density (where, bulk density of shrubs = 1.8 kg/m^3^, and herbs and grasses = 0.8 kg/m^3^ each; [35]). Aboveground biomass of seedlings and saplings were estimated using regressions based on species and height [32], [33]. Duff and litter fuel loads were estimated based on the average duff depth/stand*bulk density (bulk density of duff = 139 kg/m^3^ [32], litter = 45 kg/m^3^ [34], [35]). Fuel loads of 1, 10, 100 and 1000-hr fuels were estimated following protocols by Brown [31] [32].

#### Canopy fuel sampling and calculations

At each stand, for all trees >4 cm diameter at breast height (DBH), we recorded species, DBH, status (0 = green needles, 0.5 = yellow-green needles, 1 = red needles, 2 = no needles but 1-hr twigs present, 3 = no needles and no 1-hr twigs, 4 = dead trees not killed by MPB), tree height, crown base height (CBH) for trees of status 0–2, and crown position (dominant, co-dominant, intermediate and suppressed). We also recorded all seedlings (<1.3 m) and saplings (≤4 cm DBH) within each plot. For each tree, we recorded an effective crown base height in the field, where if an adjacent ladder fuel (sapling, seedling or other tree) could carry fire into the canopy of the tree, crown base height would be that of the ladder fuel. Sampling in 2007 did not include crown base height of status 0.5–2 trees. However, dead and dying needles and 1-hr fuels contribute to the available canopy fuel load, so crown base height of these trees is a necessary model input. Therefore, we estimated crown base height for status 0.5–2 trees sampled in 2007 based on regressions of tree height, canopy position, and DBH from 1395 status 0 trees for which crown base height was recorded in the 2007 dataset. We averaged effective crown base height for all trees with needles and 1-hr twigs for a stand estimate of canopy base height. We compared this to an estimate of canopy base height based on the lowest height in the profile where canopy bulk density exceeded 0.011 kg/m^3^, which is a standard approach that is considered unbiased, although based on an arbitrary threshold [37].

Crown fuel loads (foliage, 1-hr, 10-hr and 100-hr) were calculated for each tree/stand based on species and DBH [36]. These crown fuel loads were then reclassified as live, dead or absent based on tree status observed in the field. For example, if tree status was 1 (dead with red needles), weight of live needles and 1-hr fuels would be reclassed as dead fuel load, or if the tree status was 2 (dead with no needles), needle weight would go to zero but 1-hr fuels would be reclassed as dead.

Available crown fuel load (ACFL) was estimated based on the weight of foliage and 50% of weight of 1-hr fuels estimated to burn in crown fires, and adjusted for crown position (0.9 for codominants, 0.6 for intermediates, and 0.4 for suppressed trees; [37]). Crown bulk density was then estimated for each tree, by dividing by crown length and area sampled (kg/m^3^). According to convention, we use crown as a tree-level term, canopy as a stand-level term. Canopy bulk density (CBD) per site was estimated by assigning the crown bulk density value to each 0.25 m increment of each crown, then summing the crown bulk density values within 0.25 m increments across all trees in a stand, then averaging across stands (subplots), to produce vertical profiles of canopy bulk density for each site, following the protocols implemented in the Fire and Fuels Extension of the Forest Vegetation Simulator (FFE-FVS; [38]). The maximum value of a 3-m running mean of CBD/stand produces canopy bulk density estimates [37]. Total live and dead crown fuels by size, ACFL, CBD, crown base height and tree height were averaged across the three subsites (stands) per site sampled in 2008, while averaging across subsites was not necessary for the 2007 sites.

#### Statistical analysis

We used the R statistical programming language [39] to test for differences in field estimates of surface and canopy fuels among MPB stages via ANOVAs with pairwise comparison of means using the TukeyHSD function. We used average values of surface and canopy fuels for each MPB stage as input into a fire model to predict expected fire behavior.

#### Fire behavior modeling

Model experiments compared the expected surface and crown fire characteristics among the four MPB stages (Green, Red, Grey, Old-MPB stands) across a range of wind speeds, under three moisture scenarios (extremely dry [XD, 99%tile], very dry [VD, 95%tile] and moderately dry [D, 90%tile]). We isolated the contribution of 1000-hr surface fuels and of low available canopy fuel moistures to expected fire behavior in order to evaluate the effect of accounting for these important fuel characteristics in stands in different stages of MPB attack. All runs assume flat terrain.

To consider the effect of weather variation on fire behavior in MPB stands, we created three weather scenarios based on fuel moisture conditions during historical fires in the subalpine zone of the study area, derived from fire-weather associations in FireFamilyPlus (FFP) 4.1 (Table 1). The extremely dry [XD] scenario was based on the daily weather conditions during the five largest fires (>2000 ha) since 1985. It is notable that all these large fires occurred during a record drought year (2002), which represented the 99^th^ percentile of minimum relative humidity and maximum temperature (1985–2010), and is the driest on record in Colorado. The very dry [VD] scenario was based on weather conditions during large fires (400–2000 ha) in the 1985–2010 record, which represented the 95^th^ percentile of minimum relative humidity and maximum temperature. The moderately dry [D] scenario was based on weather conditions during small fires (399–40 ha), which reflected 90^th^ percentile conditions (Table 1).

**Table 1:** Fuel moisture inputs and effects on critical surface fire intensity.

		XD	VD	D
**A. Surface Fuel Moisture**	1-hr_dead	3	5	7
	10 hr_dead	4	6	8
	100 hr_dead	6	8	10
	1000 hr_dead	8	10	12
	Herb_live	30	35	40
	Woody_live	70	77	84
**B. Crown Foliar Moisture**	Red Foliage	3	5	7
	Green Foliage	90	100	110
**C. Available Canopy Fuel Moisture (ACFM)**	Green stage	74	82	91
	Red stage	44	50	55
	Grey stage	47	53	59
	Old-MPB stage	64	71	79
**D. Critical Surface Fireline Intensity^1^**	Green stage	921	1045	1190
^1^assumes ACFM for each stage as above	Red stage	546	627	713
	Grey stage	628	718	812
	Old-MPB stage	717	811	924
**E. Critical Surface Fireline Intensity^2^**	Green stage	921	1045	1190
^2^assumes Green-stage ACFM for all MPB stages	Red stage	991	1124	1280
	Grey stage	1063	1205	1373
	Old-MPB stage	853	967	1102

Fuel and foliar moisture values (%) and effects on critical surface fireline intensity (kW/m; the surface fire intensity needed to initial crown fire) for the extreme drought (XD), very dry (VD) and moderately dry (D) moisture scenarios: A) surface fuel moisture B) crown foliar moisture, C) average canopy foliar moisture, D) critical surface fireline intensity^1^, where canopy moisture reflects red foliage and dead 1-hr fuel moistures as shown in C, and E) critical surface fireline intensity^2^, where canopy moisture reflects that of the Green stage for all four MPB stages.

Based on these three weather scenarios, FFP estimated surface fuel moistures for dead surface fuels (1–1000 hr) and live herbaceous and woody surface fuels. We applied 1-hr surface fuel moistures to the dead (red) crown foliar moistures, which agreed under the Dry scenario with field estimates in 2010 of MPB-killed lodgepole pine needles in the study area [40]. From these estimates of moisture content of the red and green foliage in each weather scenario, for each stand we created a weighted average of the moisture content of live foliage and 50% of the live 1-hr fuels and of dead foliage and 50% of the dead 1-hr fuels [15], where foliage and 50% of the 1-hr fuels are the available canopy fuel load assumed to combust in the flaming from of the fire [37]. While BehavePlus will accommodate foliar moisture inputs 30–300%, we recognize that equations in the model are not validated for foliar moistures <60%. In BehavePlus, foliar moisture affects the transition from surface to crown fire, generally showing linear relationships with critical surface intensity, critical surface flame length, and transition to crown fire ratio (Figure S1).

Based on the low basal area of overstory trees with foliage in the Grey and Old-MPB stands, we applied wind adjustment factors in BehavePlus, which reflect expected higher wind speeds in stands with lower canopy cover (0.2 for Grey assumes 15–30% cover, and 0.15 for Old-MPB assumes 30–50% cover). We also assumed that due to the more open canopy in Grey stands, surface fuel moistures would be slightly lower (1 percentage point lower for 1, 10 and 100-hr fuels and 10 percentage points lower for live woody and live herbaceous fuels relative to expected moisture values in each scenario), similar to Page and Jenkins [13].

To characterize understory fuels, we created custom surface fuel models (here ‘model’ refers to the characteristic surface fuel loads by fuel size and type) based on field estimates of 1-hr, 10-hr, 100-hr, live herb, live woody fuel loads for each MPB stage. We used estimates of surface-area-to-volume ratios of 1-hr, live herbaceous and live woody fuels; dead fuel moisture of extinction; and heat content of live and dead fuels from the Timber Understory 1 (TU1) model, which was similar to our custom models [41].

Surface 1000-hr fuel loads varied significantly across MPB stage and can have significant influence on crown fire behavior via 1) surface fireline intensity and 2) surface fire heat per unit area. These 1000-hr surface fuels, however, are not represented in standard surface-crown operational fire models. Therefore, to account for 1000-hr fuel load effects on surface fireline intensity, we input our custom fuel model plus 1000-hr surface fuel loads for each MPB stage into the First Order Fire Effects Model (FOFEM 5.0), which predicts the mass of all measured surface fuels consumed by fire per unit area (except seedlings and saplings), under specified moisture scenarios, based on the BURNUP model [42]. We recalculated surface fireline intensity (*I*) using Byram's (1959) equation [13], based on: 1) FOFEM's expected weight of total surface fuel consumption per unit area (*w*), 2) surface spread rates (*r*) predicted from BehavePlus (which assumes that large fuels do not contribute to rate of spread of a surface fire) across a gradient of wind speeds from 10 to 100 km/hr, and 3) a constant representing low heat of combustion (*H*); where *I = Hwr*. One of the main reasons that operational fire models underpredict crown fire behavior is an inappropriate linkage between Van Wagner's [20] crown fire initiation model and Rothermel's [18] surface fire model [43]. Van Wagner's crown fire initiation model assumes that surface fire inputs include surface fuels consumed during flaming and smoldering/glowing combustion (equivalent to total surface fuel consumed estimated by FOFEM), while Rothermel's surface fire model only considers fine fuels consumed during flaming combustion. Therefore, our approach provides a more appropriate linkage between surface fire inputs and crown fire model assumptions, while accounting for 1000-hr surface fuels ignored by standard operational fire models. In BehavePlus, surface fireline intensity affects the transition to crown fire and fire type.

To account for 1000-hr fuel effects on surface heat per unit area (HPUA), we relied on Rothermel's estimates of surface HPUA, based on his Burnout model, which accounts for consumption of 1000-hr surface fuels. In standard fire models, HPUA is based only on energy release from fine fuels that affect fire spread at the flaming front, while additional energy released in the burnout phase of combustion is typically unaccounted for. We modified Rothermel's estimates of low (fuel model 10) and high (fuel model 10 plus 74 Mg/ha of 1000-hr fuels) HPUA values to reflect 1000-hr fuel loads in our MPB stands, which varied from 10 to 30 MG/ha loads of 1000-hr fuels between Green and Old-MPB stands, but did not alter HPUA according to moisture scenario. Surface HPUA affects crown fireline intensity and crown-fire flame length. For each MPB stage under each moisture scenario, we manually input the calculated: 1) surface fireline intensities expected at a 6.1 m height across a range of wind speeds from 10 to 100 km/hr, and 2) surface HPUA values to initiate the crown fire module in BehavePlus.

Among the MPB stages under three weather scenarios, we compared: 1) surface fireline intensity, 2) critical surface fireline intensity, 3) transition ratio, 4) active ratio, 5) crown fireline intensity, and 6) the minimum wind speed at which four crown-fire types were expected to occur. The transition from surface to crown fire is based on a ratio of surface fireline intensity to the critical surface fireline intensity, where a transition ratio >1, indicates the wind speed at which crown fire is possible, with ratios significantly >1 generally indicating a higher likelihood. After transition to crown fire, the fire may become: 1) a passive crown fire torching individual trees then dropping back to a surface fire, 2) an active crown fire spreading through the overstory crowns, or 3) a conditional crown fire, where it does not transition to crown fire within the stand, but would sustain an active fire entering the stand. The occurrence of these fire types can be predicted based on the combination of the wind speeds at which the transition and the active ratios are >1 (Table 2). The active ratio is derived from crown fire rate of spread (related to surface fuel moisture and 6.1-m wind speed) divided by the critical crown fire rate of spread (related to canopy bulk density). When the active ratio is >1, an active crown fire is possible, with higher values generally indicating a higher probability of active crowning.

**Table 2:** Boolean logic for predicting fire types.

		Active Fire?	
		NO	YES
**Transition to**	NO	Surface	Conditional Crown
**Crown Fire?**	YES	Torching	Active Crown

Predicted fire types based on the wind speed at which the Transition Ratio and Active Ratio are <1 (NO) and/or >1(YES). If the Transition Ratio is ≥1 for a given wind speed, Surface Fireline Intensity is sufficient for transition to crown fire at that wind speed or greater. If the active ratio is ≥1 for a given wind speed, the fire is predicted to be an active crown fire at that wind speed or greater. Fire types are: 1) Surface (understory fire that does not reach the crowns), 2) Torching (also known as Passive Crown Fire; surface fire with occasional torching of individual trees), 3) Conditional Crown (active crown fire possible, if the fire transitions to the overstory, but such crown transition is not predicted in the current stand for the given wind conditions), and 4) Active Crown Fire (fire spreads from crown to crown).

## Results

**Figure 3:**
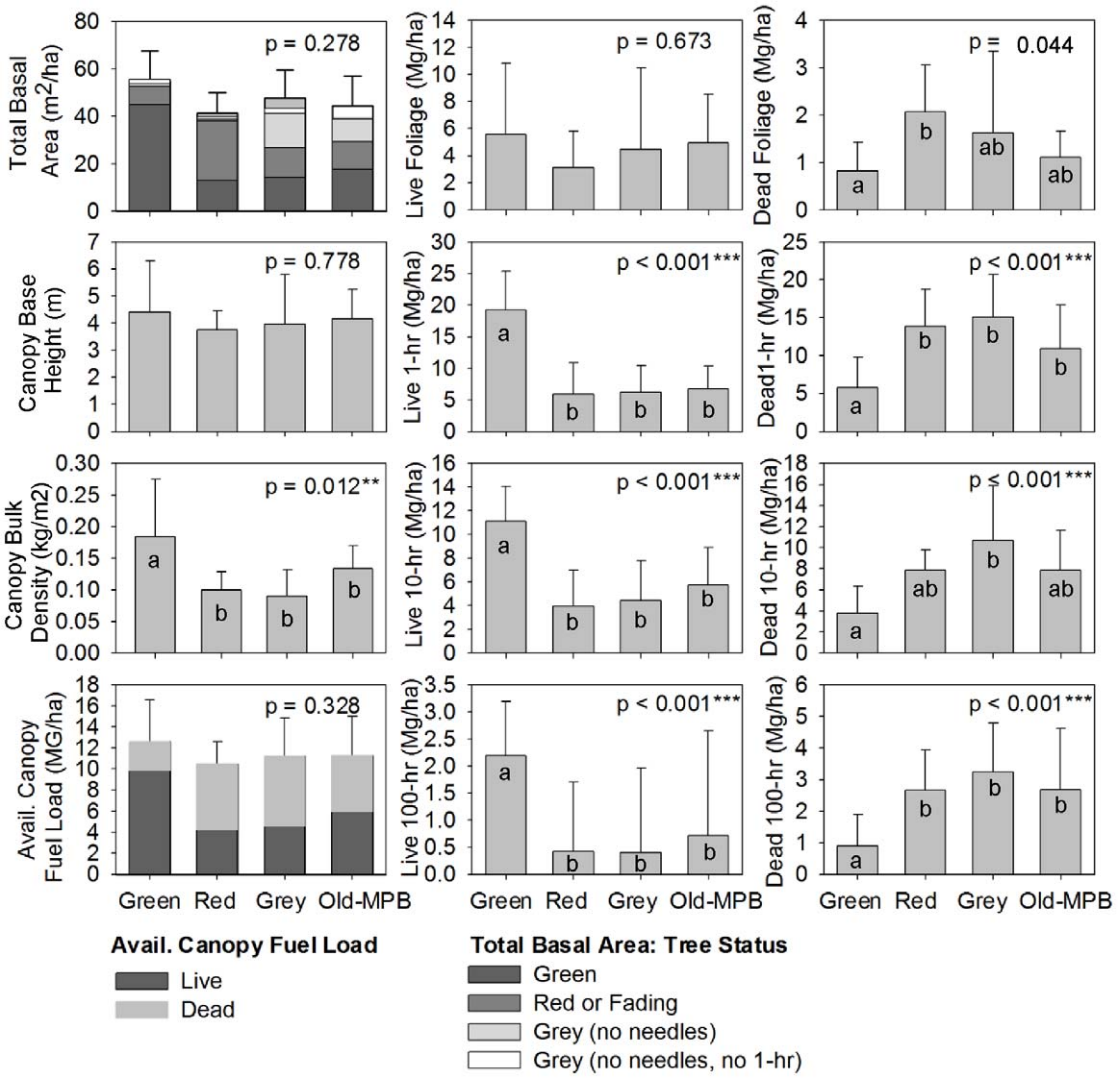
Dead surface fuels in four stages of MPB attack. Comparison of average dead surface fuel loads among four stages of MPB attack (Green, Red, Grey, Old-MPB; see text for description of MPB stages), with bars representing standard errors. P-values from ANOVAs in upper right of each graph, with letters indicating significant difference based on Tukey's pairwise comparison of means.

Average basal area of tree classes confirmed the assumed characteristics of our MPB stages. While total basal area was not significantly different across the MPB stages (ave. = 45 m^2^/ha; *p* = 0.278, Figure 3), live basal area was significantly higher in the Green stands compared to other stands (45 vs. 15 m^2^/ha; p<0.001). Basal area of red and fading trees was highest in the Red stands compared to other stands (25 vs. 9 m^2^/ha; p<0.001). Basal area of grey trees with no needles was significantly higher in Grey stands (14 m^2^/ha, compared to 9 m^2^/ha in Old-MPB stands, and 0.65 m^2^/ha in Green and Red stands, on average; *p* = 0.003). Basal area of grey trees with no needles and no 1-hr fuels was highest in Old-MPB stands, but not significantly different from other MPB stages (*p* = 0.159). The percentage of green trees was 85% in Green stands, 58% in Red stands, 49% in Grey stands, and 64% in Old-MPB stands. Red and fading trees comprised 32% of the Red stands, and 15% of the Grey stands. Lodgepole pine trees were 75% of all trees in the Green, Red and Grey stands, on average, and 63% in the Old-MPB stands, where MPB had killed many of the host trees. Average tree density was 1691 trees/ha. Saplings were relatively sparse (78 saplings/ha) with no significant differences among MPB stages (p = 0.211). While seedling density was higher (1919 seedlings/ha) it was not significantly different among MPB stages (p = 0.850; Figure S2).

Analysis of the collected cores indicated that the age of Old-MPB stands ranged from 125–300 yrs old. Growth release data showed that prior MPB-attacks in the Old-MPB stands dated to the late 1970s to early 1980s in nine stands, and to 1958 in one stand (mean = 1980, SD = 6.4 yrs, range = 1958–83).

### Surface fuel loads

**Figure 4:**
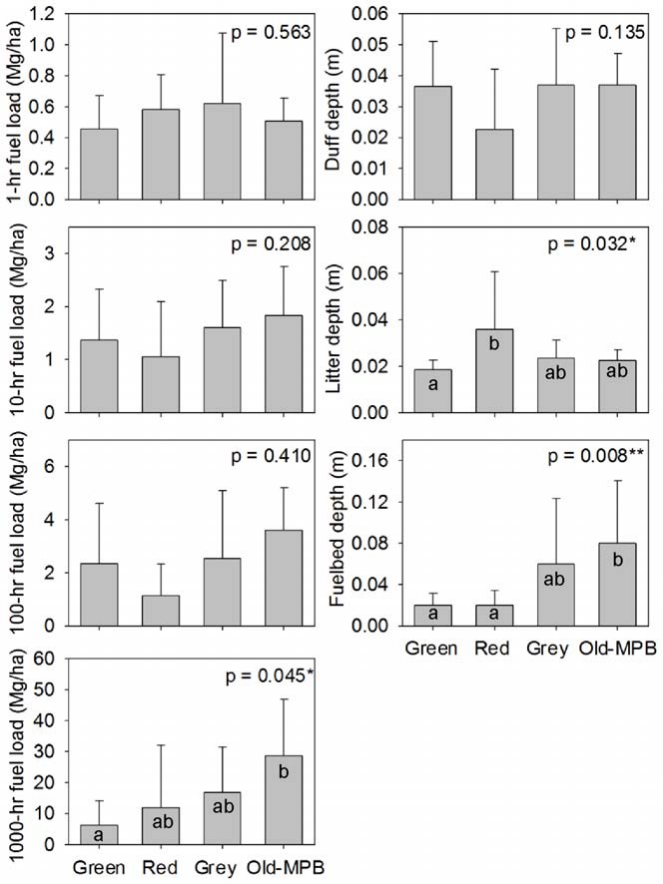
Canopy fuels in four stages of MPB attack. Total and proportion of total basal area of green, red/fading, and grey trees among four stages of MPB attack (Green, Red, Grey, Old-MPB; see text for description of MPB stages), and comparison of average canopy fuel loads among the four MPB stages of MPB attack with bars representing standard errors. P-values from ANOVAs in upper right of each graph, with letters indicating significant difference based on Tukey's pairwise comparison of means.

MPB stages exhibited significant variation in some surface fuels (Figure 4). Thousand-hour fuels were significantly higher in the Old-MPB stands compared to the Green stands (*p* = 0.045), while fuelbed depth was significantly higher in the Old-MPB stage compared to the Green and Red stages (*p* = 0.008). However, 1, 10, and 100-hr fuels were not significantly different among MPB stages. Litter depth was significantly higher in the Red stands, almost twice as deep as in the Green stands (*p* = 0.032), reflecting significant accumulation of needles dropped from recently attacked trees. There were no significant differences in surface fuel loads of live or dead shrubs, herbs, grasses or live seedlings and saplings among MPB stages (Figure S2).

### Canopy fuel loads

In terms of canopy fuels, dead foliage fuel load was significantly higher in the Red stands, and lower in the Green stands (*p* = 0.044; Figure 3). Live 1-hr, 10-hr and 100-hr canopy fuels were significantly higher in Green stands, while dead 1-hr and 100-hr canopy fuels were significantly lower in Green stands. Dead 10-hr fuels were significantly lower in the Green stage compared to the Grey stage. Canopy bulk density was significantly different among MPB stages (*p* = 0.012) with Red, Grey, and Old-MPB stands significantly lower than Green stands, but canopy base height did not vary across MPB stages, which are two important variables in modeling crown fire behavior. Sapling and seedling fuel loads were very low (0.03 Mg/ha and 0.136 Mg/ha, respectively, on average) compared to available fuel loads of canopy trees (11 Mg/ha), and did not significantly vary among MPB stages (*p* = 0.759; Figure S1).

### Fire behavior modeling

#### Surface fire intensity

**Figure 5:**
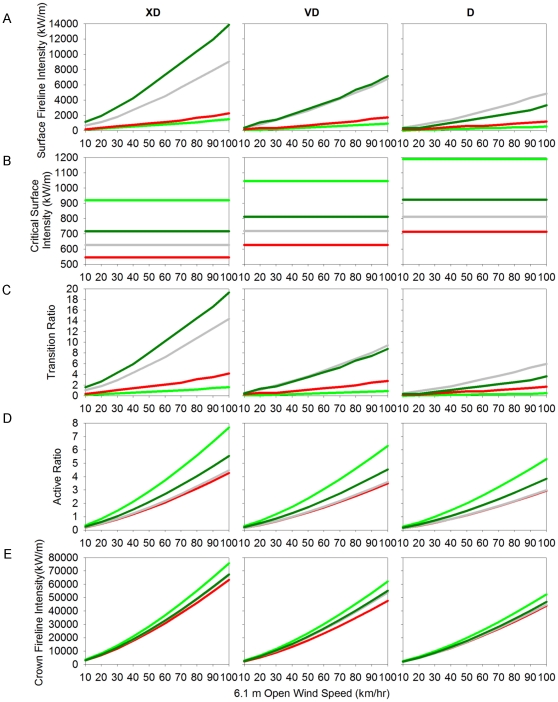
Surface and crown fire outputs modeled for four stages of MPB attack. Comparison of predicted fire behavior: A) Surface Fireline Intensity, B) Critical Surface Intensity, C) Transition Ratio, D) Active Ratio, E) Crown Fireline Intensity for Green (light green line), Red (red line), Grey (grey line), and Old-MPB (dark green line) stages under Extreme Drought (XD), Very Dry (VD) and Moderately Dry (D) moisture scenarios (see text for description of MPB stages and moisture scenarios).

Surface fireline intensity was highest for the Old-MPB and Grey stands under all moisture scenarios (Figure 5A). Red and Green Stands had comparatively lower surface fireline intensity, reflecting lower 1000-hr surface fuel loads in these stands. Differences were greatest under the extremely dry weather scenario. Under the moderate moisture scenario, differences in surface fireline intensity among MPB stages were less marked; with lower consumption of 1000-hr fuels, however, Grey stands showed a slightly higher surface fireline intensity compared to Old-MPB stands, due to a more open canopy in Grey stands and associated higher surface spread rate.

Surface fireline intensities reported above, which incorporate consumption of 1–1000 hr surface fuels, were on average 2.5 times higher (4 times higher for the Old-MPB class) compared to the consumption model without 1000-hr fuels incorporated, and on average 15 times higher (28 times higher for the Old-MPB class), compared to standard surface fire assumptions that only account for flaming front combustion of fine fuels (no consumption of fuels or 1000-hr surface fuel loads), which are standard assumptions in NEXUS and BehavePlus. This suggests that exclusion of existing 1000-hr fuel loads from the models, when in fact they are present, such as in old-MPB stands, can result in underestimated surface fireline intensity in standard surface fire models, which can strongly influence our understanding of the probability of transition to crown fire.

#### Transition to crown fire

Critical surface intensity, the surface fireline intensity required for transition to a crown fire, was highly influenced by low canopy moisture (≤60%), which varied considerably across MPB stages under different moisture scenarios (Table 1C, D; Figure 5B). In general, critical surface fire intensity was positively correlated with canopy foliar moistures within a moisture scenario, meaning less moisture in the canopy results in lower surface intensity needed for crowning. Therefore, Red stands were more likely to crown under lower surface fire intensities, compared to other MPB stages. Green stands with higher canopy moistures exhibited much higher critical surface intensities (1190-921 kW/m under D-XD moisture scenarios) compared to Old-MPB (924-717 kW/m), Grey (812-628 kW/m) and Red (713-546 kW/m). In general, as conditions become drier, critical surface intensity declined with less variation among MPB stands.

When available canopy fuel moisture was held constant, reflecting that of Green stands in each moisture scenario, critical surface fireline intensities were about 25% higher on average (1092 vs. 804 kW/m) and were more similar among MPB stands (range: 520 vs. 644 kW/m), reflecting minor differences in canopy base height across stands (Table 1E). This suggests that accounting for low canopy foliar moisture is an important aspect of modeling fire behavior in stands with significant MPB mortality.

Transition ratio, which indicates the likelihood of transition to crown fire, reflected trends in surface fireline intensities (Figure 5C). The transition ratio was very high under the extreme drought scenario in Old-MPB and Grey stands, where crown fire was expected ≥10 km/hr wind speeds, while crown fire was expected in Red and Green stands at wind speeds ≥30 km/hr and ≥65 km/hr, respectively. Under the moderate weather scenario, Grey stands were predicted to crown first at 20 km/hr (due to the higher spread rate in more open stands), Old-MPB stands would crown at 40 km/hr, Red stands at 68 km/hr, while the transition ratio for Green stands indicated that surface fireline intensity was not sufficient for transition to crown fire below 100 km/hr.

Low canopy moistures in Red stands made crown transition more likely at lower wind speeds compared to simulations where foliar moisture was held constant at Green stand values across all MPB stages. Specifically, the effect of variation in foliar moistures made crowning likely at wind speeds 30 km/hr lower in the Red stands, yet with little effect on crowning winds speeds in Grey and Old MPB stands as red foliage is no longer present.

#### Crown fire type

Active crown fires were likely to occur at lower wind speeds in the Red, Grey and Old-MPB stands compared with Green stands (Figure 5D, Table 3), and minimum wind speeds for active crown fire in Grey and Old-MPB stands were less sensitive to moisture scenario compared to that for Green and Red stages (Table 3). However, given that conditional crown fires are possible in Green stands at relatively low wind speeds (∼30–40 km/hr), the predicted differences in crown fire wind speeds may not result in significantly different fire behavior under windy, and especially gusty burning conditions typical of large fires in this zone. In general, low canopy foliar moistures due to MPB mortality made active fire more likely in Red stands at lower wind speeds (40 km/hr vs. 60 km/hr), however, there was no change in the expected fire type occurrence for Grey and Old-MPB stands where foliage is absent and the fire type seems highly influenced instead by high surface fuel loads (Table 3, 4).

**Table 3:** Wind-speed thresholds for predicted fire types (km/hr), where canopy fuel moisture varies.

		XD	VD	D
Green stage	Surface	<30	<30	<40
	Torching			
	Conditional Crown	30–65	30–100+	40–100+
	Active Crown	>65	>100+	>100+
Red stage	Surface	<30	<50	<55
	Torching	30–40	50–55	
	Conditional Crown			55–70
	Active Crown	>40	>55	>70
Grey stage	Surface	<10	<20	<30
	Torching	10–40	20–55	30–55
	Conditional Crown			
	Active Crown	>40	>55	>55
Old-MPB stage	Surface	<10	<20	<40
	Torching	10–30	20–40	40–45
	Conditional Crown			
	Active Crown	>30	>40	>45

Wind speeds (km/hr) at which four fire types are expected for the four MPB stages: Green, Red, Grey and Old-MPB under three moisture scenarios: extreme drought (XD), very dry (VD) and moderately dry (D) moisture scenarios, where available canopy fuel moisture reflects the proportion of red and green needles in each stage as in Table 1C. See Table 2 for description of fire types.

**Table 4:** Wind-speed thresholds for predicted fire types (km/hr), where canopy fuel moisture is constant.

		XD	VD	D
Green stage	Surface	<30	<30	<40
	Torching			
	Conditional Crown	30–65	30–100+	40–100+
	Active Crown	>65	>100+	>100+
Red stage	Surface	<40	<55	<55
	Torching			
	Conditional Crown	40–60	55–80	55–100+
	Active Crown	>60	>80	>100+
Grey stage	Surface	<20	<30	<40
	Torching	20–40	30–55	40–55
	Conditional Crown			
	Active Crown	>40	>55	>55
Old-MPB stage	Surface	<10	<20	<45
	Torching	10–30	20–40	45–50
	Conditional Crown			
	Active Crown	>30	>40	>50

Wind speeds (km/hr) at which four fire types are expected for the four MPB stages: Green, Red, Grey and Old-MPB under three moisture scenarios: extreme drought (XD), very dry (VD) and moderately dry (D) moisture scenarios, where available canopy fuel moisture is held constant across MPB stage at that of the Green stage. See Table 2 for description of fire types.

#### Crown fire intensity

Crown fireline intensity is calculated from the surface heat per unit area (positively correlated with 1000-hr fuel load), crown fire heat per unit area, and crown rate of spread (a function of surface fuel moistures) and is used to calculate crown flame length [19]. Crown fireline intensities were relatively similar among MPB stands (e.g. 13% difference at 60 km/hr wind speeds under the extreme drought scenario), with Green stands having the highest intensities, Red stands the lowest, Grey and Old-MPB predicted to burn at intermediate intensities (Figure 5E). Crown fireline intensities increased with extreme drought conditions (on average by 30% compared to the moderate weather scenario), showing higher sensitivity to moisture conditions than variation in fuel complexes among MPB stages.

## Discussion

The proportion of trees killed by MPB plays an important role in potential fire behavior following MPB outbreaks [14]. We found that after more than a decade following the onset of MPB outbreak in north-central Colorado, only about 50% of the trees and 70% of the basal area was dead in lodgepole pine stands in the Red and Grey stages, consistent with findings by Simard et al. [15] and Klutsch et al. [44]. The Old-MPB stands reflect a MPB outbreak predominantly from the 1980s, which was much less severe than the current outbreak [24], and therefore may not be an adequate analogue for fuels 30 yrs after the more severe current outbreak, although is indicative of expected trends in fire behavior relative to fuel configuration during this stage. From aerial detection surveys in British Columbia, Kurz et al. [45] estimated that moderate-severe mortality (defined as 30–50% biomass killed) affected only about one-third of the outbreak extent. These studies indicate that even severe outbreaks do not result in 100% tree mortality. Furthermore, we found that each MPB stage had trees in all phases of attack, which, in addition to spatial-temporal variation in the distribution of MPB stages across the landscape, has important implications for fire behavior, forest regeneration, and carbon storage.

### Surface fuels and surface fire behavior

The only surface fuels that varied significantly across MPB stages in lodgepole pine stands in Colorado were surface litter and large-diameter fuels. Surface litter, due to fall of dead canopy foliage, was twice as high in Red stands compared to Green stands, similar to significant litter accumulations noted in other studies [15], [44], [46]. However, this spike in needlefall contributed little (≤10%) to predicted surface fireline intensities in Red stands.

Old-MPB stands had significantly higher loads of large-diameter (1000-hr) surface fuels compared to Green stands, similar to other field studies [15], [44], [46]. When burned, large surface fuels contribute to higher surface fire intensity and higher surface heat release per unit area that can significantly affect crown fire behavior [19]. In our approach to calculating surface fireline intensity, we considered fine fuels consumed during flaming combustion and those larger fuels consumed during subsequent combustion; in estimating surface heat per unit area, we considered additional energy released in the burnout phase of combustion of larger fuels. While this approach may represent an upper bound of the potential effect of large surface fuels on crown fire behavior, it is known that combustion of large-diameter surface fuels can have significant impacts on crown fire development. Our model runs predicted active crown fires under reasonable wind speeds, which is expected behavior in lodgepole pine systems, but which many other modeling efforts have been unable to achieve. Standard operational fuel models (e.g. BehavePlus, NEXUS, FVS-FFE), may need to be adjusted to account for 1000-hr surface fuel load effects on surface fireline intensity or heat per unit area, to better portray their influence on potential transition to crown fire. Explicitly accounting for the contribution of 1000-hr surface fuels to fire behavior, we found that surface fireline intensities were highest for Old-MPB and Grey stands due to a combination of high 1000-hr fuel loads and assumed higher wind speeds under more-open canopies due to tree mortality from MPB. High surface fireline intensities strongly influence the probability of crowning, as indicated by the high transition ratios for Old-MPB and Grey stands.

While large-diameter surface fuel loads were high during the roughly 5–30+ yrs following MPB attack, surface fuel loads are generally high in lodgepole pine forests in Colorado as a consequence of self-thinning and other disturbance events such as severe fire and blowdown [47], [48], [49]. Dense post-fire stands of lodgepole pine in Wyoming created significant loads of large surface fuels by self-thinning over 50 yrs [50], although self-thinned trees would be smaller in diameter than those killed by MPB. Severe stand-replacing fires release considerable loads of large surface fuels in the short term due to post-fire fall of burned trees, likely higher loads than caused by MPB where mortality is seldom 100%. For example, lodgepole pine stands sampled a decade after the 1988 fires in Wyoming recorded 170 Mg/ha in dead wood (50 Mg/ha was downed wood, 120 Mg/ha was standing snags yet to fall; [51]). A blowdown event in a subalpine forest in Colorado contributed high surface fuel loads that varied considerably across a 0–100% mortality gradient (0—500 Mg/ha; [52]). Klutsch et al. (2011) predicted that surface fuel loads due to 80% treefall from the current MPB outbreak would be within historical ranges found in lodgepole pine forests [53]. Although MPB-induced loads of large surface fuel loads are high relative to unaffected stands, such loads are not uncharacteristic of lodgepole pine stands.

Managers are often concerned about the effect of accumulated 1000-hr surface fuel loads on soil heating and stand recovery, if burned. While no studies to date have explicitly tested these responses in old-MPB stands, modeled fire residence times and maximum temperatures at the mineral soil surface increased while seedling establishment significantly declined in a post-fire study across a 0—500 Mg/ha surface fuel load gradient resulting from a subalpine forest blowdown [52]. Our study and Pelz's [54] estimate surface fuel loads 30-yrs post-MPB were only 30 and 60 Mg/ha respectively, which reflect expected low soil heating and high post-fire regeneration, comparatively. Additional evidence, from severe fires in Yellowstone that burned areas which had burned 10–50 yrs previously and therefore where surface fuel loads from mortality due to the previous fire would have been high, showed that understory cover and tree seedling establishment were relatively high [55]. Duff moisture plays an important role in determining mineral soil heating under burning and smoldering slash piles [56]. These studies suggest that although surface fuel loads are relatively high in Grey and Old-MPB stages, soil heating and post-fire recovery may not be significantly affected in the decades following moderate levels (<50%) of MPB mortality, although more explicit research is needed.

### Canopy fuels and crown fire behavior

Transition from surface to crown fire is determined by the critical surface fireline intensity needed to ignite canopies of: 1) specified height, and 2) moisture content (which in this study is the moisture content of the ‘available canopy fuel load’ [ACFL] that burns in the flaming front, where ACFL is assumed to be 50% of 1-hr fuels and all foliage). If surface fire intensity exceeds that critical threshold, either a passive (torching) or active canopy fire will burn. Active crown fire will occur when a critical crown fire spread rate (based on surface fuel moisture and effective wind speed), is reached that will sustain burning in canopies of a certain bulk density. Conditional crown fire behavior occurs when canopy bulk density is sufficient for active crown fire, but flame lengths are too low or canopy base height or foliar moisture are too high for crown fire initiation [57].

Although canopy base height plays an important role in initiating crown fire, average effective height of the base of the crowns did not vary with MPB stage in this study, similar to estimates by Simard et al. 2011. While standard fuel models do not typically account for saplings and seedlings in surface or canopy fuel inputs, we accounted for their role as ladder fuels by recording their potential lowering of effective crown base height estimates in the field. Furthermore, these understory tree densities and fuel loads were relatively low and similar across MPB stage.

We used a fairly conservative estimate of canopy base height, relying on a stand average of effective crown base heights estimated in the field for each tree in the stand (∼4 m) rather than the height at which a threshold canopy bulk density is attained in a stand (varies from 0.011 kg/m^3^ to 0.320 kg/m^3^ in the literature). Estimating canopy base height using Scott and Reinhardt's [57] standard threshold (0.011 kg m^3^) would have decreased canopy base height in our study (from 4 m to 1.25 m), which would have been considerably lower than similar studies, and would have contributed to much higher probabilities of crown fire in our study (i.e. lower critical surface fire intensity and lower wind speeds needed for active crown fire), and likely would have masked any differences in fire behavior between MPB stages. Nonetheless, average estimates of canopy base height likely do not adequately represent fire behavior within a stand, where the lowest individual crowns may initiate torching within a stand, after which active crown fire may subsequently propagate. Therefore, relying on average crown base height likely overerestimates the height of the effective canopy base, and may present an underprediction of crowning potential.

Available canopy fuel moisture content, in contrast, varied considerably across MPB stage, with moisture content of Red and Grey stands dipping to about one-third less than that of Green stands, in each moisture scenario. While individual red needles and dead 1-hr fuels have very low fuel moistures (5–15%) compared to green needles (90–120%; [12]), Red and Grey stands with about 50% mortality had available canopy fuel moisture of about 45% under the extreme weather scenario, based on a weighted average of live and dead available canopy fuels (Table 1). Live trees also have dead fuels in their crowns, about 18% of the live crown weight on average in this study (based on Brown's [36] equations), which also decreased available canopy fuel moistures in green stands. Dry foliage and small branches have been shown to ignite more readily in lab experiments, therefore red trees with high crown base height may ignite unexpectedly compared to similar green trees with easier fire spread between red tree crowns [12], [58]. Single tree model simulations suggest almost a doubling of the total net heat release from a red-needle tree compared to a green-needle tree [12], while lower foliar moisture may increase crown consumption by fire. While fire may not respond to average canopy foliar moisture conditions as modeled, our results showed that Red and Grey stands were more likely to passively crown under lower surface fireline intensities, while Green stands required the higher surface fireline intensities or lower canopy base height in order to torch, consistent with lab experiments and experimental burns in stands with simulated MPB-mortality [59].

No other operational modeling studies have accounted for low (<70%) available canopy fuel moisture content in stands affected by MPB, however, the Canadian Forest Service Fire Behaviour Program (CFS-FBP) does recognize fuel types for dead (insect killed) balsam fir stands, which reflect drier canopy and fallen fuels. BehavePlus is the only operational model that allows foliar moistures inputs <70%. While low values remain to be validated in these models, where canopies are assumed to be green, effects of low canopy fuel moisture on critical surface fireline intensity, critical surface flame length, and transition ratio are additive and transparent (Figure S1), and resulted in more realistic crown fire behavior than other studies.

Furthermore, higher crown flammability due to low moisture content overwhelmed the influence of lower canopy fuel continuity in Red and Grey stands, which if considered alone would predict that active fires would be less likely during these stages. We observed almost 50% lower canopy bulk density in Red stands compared to the Green stands, similar to Simard et al. [15], yet active fires were likely at wind speeds 25 km/hr lower in Red stands compared to Green stands. Most studies to date indicate a decline in active crown fire potential during the Red phase, due to limitations in modeling low foliar moistures [13], [15], [60]. Interestingly, however, results from a physics-based fire model indicate that increased crown fire potential in the Red phase is dependent upon the fire intensity generated by the pre-outbreak surface fuels, so that when surface fire intensity was high, the extent of red trees in the stand did not affect crown fire hazard [14].

In Grey stands, the combination of lower canopy fuel moistures, higher penetration of wind through the stand, and higher surface fireline intensity resulting from MPB-induced treefall resulted in active fires more likely at wind speeds 25 km/hr lower than the wind threshold for Green stands. While we expected active crown fires to burn more easily in Red stands, past modeling and expert opinion indicated Grey stands would be less prone to active crown fire, not more. Differential accounting for effects of high surface fire intensities and low foliar moistures may explain discrepancies between our modeling outcome and others' [14] [16], where active crown fire potential was predicted to be relatively low during the Grey phase.

In Old-MPB stands, we witnessed 30% lower canopy bulk density compared to Green stands, similar to Page and Jenkins [46]. Studies to date have hypothesized that active crown fire would be more likely in Old-MPB stands primarily due to a reduction in canopy base height (which varied little across MPB stage in this study), not due to the contribution of high surface intensities from heavy loads of large surface fuels from MPB-induced treefall.

Indeed, many of the US models (NEXUS, FlamMap, BehavePlus, FARSITE, FFE-FVS, and FMAPlus) underpredict potential crown fire behavior in conifer forests of western North America, where unrealistically high wind speeds are required for the onset of crowning and active crown fire propagation, indicating behavior inconsistent with documented wildfires [43]. For example, Nexus predicted Green stands in Yellowstone would not transition to crown fire under extreme drought, except with unrealistically high winds exceeding 600 km/hr; [15]), while crown fire is in fact the characteristic fire behavior in green lodgepole pine stands under extremely dry windy conditions. A primary reason for underprediction of crown fire probability in most models is low surface fireline intensities, which confer a low potential for crown fire initiation. Our study explicitly overcomes this by calculating surface fireline intensity outside the model. This not only allowed us to account for 1000-hr surface fuels ignored in standard models, but also to use Byram's equation for surface fireline intensity rather than Rothermel's, which is consistently lower. While standard fire models link Rothermel's [18] surface fire model for rate of spread and intensity and the Van Wagner's [20] crown fire initiation model, the latter model was actually developed for Byram's [61] fireline intensity equations [43]. Plus, we accounted for surface fuels consumed during flaming and smoldering/glowing combustion, which provides a more appropriate linkage between surface fire inputs and crown fire model assumptions. These model mismatches contribute to an inherent underprediction of potential for crown fire initiation. Another shortcoming of the standard operational models is that they apply a reduction factor to the predicted crown-fire rate of spread, termed crown fraction burned, which is a modeling construct absent from BehavePlus. Lastly, saplings and seedlings are not formally recognized by operational fire models, so in instances where their role as ladder fuels and overall fuel load are high, their lack of representation may contribute to underestimation of crown fire occurrence. Overall, US models appear to consistently underpredict crown fire behavior, especially in stands with fuel complexes related to MPB mortality where the effects of low foliar moistures and 1000-hr surface fuels are not explicitly modeled [43].

### Observed fire behavior following past bark beetle activity

Two retrospective studies assessed severity of 2002 fires in Colorado (based on Differenced Normalized Burn Ratio [dNBR]) in areas where prior bark beetle outbreaks (1–5 and 60 yrs prior to fire) and blowdown had occurred. Results showed that old outbreaks and blowdown had significant effects on subsequent fire severity, while the recent (<5 yrs prior) beetle activity did not [62], [63]. In Yellowstone National Park, Turner et al. [64] found that severe MPB mortality (7–15 year prior to fire) also increased the probability of subsequent severe crown fire in 1988. While the models we employed do not predict severity per se (which reflects the lethality of the fire or simply loss of organic matter), surface fireline intensity (heat output per unit area) was highest in Grey and Old-MPB stands, where crown fire was more likely under lower wind speeds, consistent with conclusions about a lasting signal of MPB on fire severity in these studies.

In terms of the probability of fire occurrence (fire risk), results from retrospective studies are more ambiguous. Lynch et al. [65] found that severe fires of 1988 in Yellowstone National Park were more likely to occur in lodgepole pine forests with MPB activity 15 yrs prior, but not 7 yrs prior. However, in Colorado Bebi et al. [66] found that small fires were not more likely to burn spruce-fir forests affected by previous spruce beetle outbreak (10–50 yrs prior to subsequent fires). Kulakowski and Jarvis [67] found no detectable increase in the occurrence of high-severity fires following MPB outbreaks.

In sum, modeling the effect of MPB-induced changes in fuel complexes on potential fire behavior is challenging, given the need for models to better account for factors such as the presence of large surface fuels and effects of dead canopy fuels, the variability in fuels within a stand, and potential non-linear relationships among fuels, wind and fire behavior. Empirically, we witnessed only about 50% mortality of the trees due to MPB attack in the Red and Grey stands, with high variability in the field in each of the four MPB stages considered, contrary to an idealized trajectory of synchronous change in fuels following MPB attack (e.g. Figure 2). Although fine fuel accumulation following tree death occurs naturally, it is variable temporally and spatially as not all trees succumb simultaneously, even at a small scale. In addition, the rate of accumulation of large surface fuels is non-linear, likely reflecting stochastic snow and wind events [68], [69]. Such variation in surface fuel loads is naturally high in lodgepole pine-dominated forests, however, independent of MPB effects. In addition, level of mortality and variation in pre-fire stand composition and structure has been shown to have a significant effect on predicted fire behavior [14], [16].

By explicitly accounting for the contribution of 1000-hr surface fuel loads to surface fireline intensity and crowning probability, and the potential effect of low (<70%) canopy foliar moistures on crown fire behavior, our modeling approach produced more realistic fire behavior predictions for lodgepole pine forests, where active crown fire was likely in Green stands under extreme drought and realistic wind conditions, unlike previous operational modeling efforts. Overall, active fire is more likely to occur at lower wind speeds in Red and Grey stands because of lower canopy fuel moisture conditions. Surface fire intensity is expected to be higher in Grey and Old-MPB stands, keeping active fire risk high under less extreme moisture conditions. While incredibly difficult to fight tactically due to obstacles created by downed wood and extreme behavior, active crown fire under extreme burning conditions is characteristic of lodgepole pine forests, however, which are expected to be resilient to severe fires burning a variety of fuel complexes from sometimes successive disturbance events.

## Supporting Information

Figure S1 **Canopy foliar moisture effects on fire model output.** Effect of variation (30%–90%) in canopy foliar moisture (a.k.a. available canopy fuel moisture) on critical surface intensity, critical surface flame length, and transition ratio (where when >1 indicates probable transition from surface to crown fire (passive, active or conditional crown fire), which are the three fire behavior outputs that are directly related to canopy foliar moisture.
(DOCX)

Figure S2 **Live surface fuels in four stages of MPB attack.** Comparison of average live surface fuel loads among four stages of MPB attack (Green, Red, Grey, Old-MPB), with bars representing standard errors. P-values from ANOVAs in upper right of each graph.
(DOCX)
