# Supplementary material for: Effects of Mountain Pine Beetle on Fuels and Expected Fire Behavior in Lodgepole Pine Forests, Colorado, USA
Source: PLoS One. 2012 Jan 17;7(1):e30002. doi: 10.1371/journal.pone.0030002 (PMC3260208; doi:10.1371/journal.pone.0030002)
Supplement: Figure S2 — Live surface fuels in four stages of MPB attack. Comparison of average live surface fuel loads among four stages of MPB attack (Green, Red, Grey, Old-MPB), with bars representing standard errors. P-values from ANOVAs in upper right of each graph. (DOCX) [file pone.0030002.s002.docx]

Figure S1.

Green Red Grey Old-MPB

Green Red Grey Old-MPB

Green Red Grey Old-MPB

p = 0.759

p = 0.249

p = 0.614

p = 0.239

p = 0.686

p = 0.619

p = 0.704

p = 0.894

p = 0.155

p = 0.759

p = 0.747
